# Concurrent MRI findings of Dupuytren’s disease and A1 pulley stenosing tenosynovitis: A case report

**DOI:** 10.1016/j.radcr.2026.05.042

**Published:** 2026-06-12

**Authors:** Scott D. Wuertzer, Balamurugan Ulaganathan, Murat Acar

**Affiliations:** Department of Radiology, Wake Forest School of Medicine, Winston-Salem, NC, USA

**Keywords:** Dupuytren’s disease, Trigger finger, MRI

## Abstract

Dupuytren's disease and trigger finger are common fibroproliferative disorders that may coexist clinically. Although their clinical association has been previously described, reports emphasizing simultaneous MRI findings of both entities are limited. We describe a 51-year-old woman with progressive hand stiffness and digital triggering. MRI demonstrated low-signal palmar cord-like thickening with marked enhancement, consistent with early Dupuytren’s disease, along with thickening and enhancement of the A1 pulley and associated flexor tendon sheath changes compatible with stenosing tenosynovitis. This case highlights the utility of MRI in evaluating multifactorial hand symptoms and demonstrates simultaneous imaging features of two distinct but potentially related fibroproliferative processes.

## Introduction

Dupuytren’s disease is a chronic fibroproliferative disorder of the palmar fascia characterized by nodule and cord formation that may progress to digital flexion contractures [1]. Trigger finger, or stenosing tenosynovitis, results from thickening of the flexor tendon sheath and constriction at the A1 pulley, leading to painful catching or locking during finger motion [2,3].

Although these conditions involve distinct anatomic structures, they share epidemiologic risk factors, particularly diabetes mellitus, and may coexist. Prior clinical studies have suggested an association between the two disorders, raising the possibility of overlapping fibroproliferative mechanisms; however, the exact nature and magnitude of this relationship remain uncertain [4]. Furthermore, while several reports have described the MRI features of Dupuytren’s disease and trigger finger individually [5,6]. However, descriptions focusing on their concurrent MRI appearance in the same patient appear to be limited. MRI provides detailed soft-tissue characterization of the palmar fascia, flexor tendons, and pulley system, and may be useful when multiple pathologies are suspected.

We report a case demonstrating simultaneous MRI features of Dupuytren’s disease and stenosing tenosynovitis in the same patient.

## Case report

A 51-year-old woman presented with progressive bilateral hand stiffness and pain of gradual onset, without trauma. She had a history of diabetes mellitus. Symptoms were more pronounced in the right hand, particularly with intermittent triggering and locking of the thumb over the preceding three months, with pain rated 8/10.

On examination, sensation was intact in the median, ulnar, and radial nerve distributions. The tabletop test was negative, and she could form a composite fist. Tenderness was present over the A1 pulley regions of the thumb, index, middle, ring, and little fingers. Triggering was elicited in the right thumb. Palpable nodules were noted in the mid-palmar region between the distal and proximal palmar creases.

Radiographs of the right hand showed no acute osseous abnormality or joint space narrowing.

Although clinical examination and ultrasound are commonly sufficient for the diagnosis of Dupuytren’s disease and trigger finger, MRI was obtained to evaluate for possible underlying inflammatory synovitis in the setting of persistent bilateral hand stiffness despite treatment. The patient had prolonged morning stiffness and clinical concern for inflammatory arthritis; however, laboratory studies, inflammatory markers, and radiographs were unrevealing, and no definite synovitis was identified on physical examination. Therefore, contrast-enhanced MRI of the right hand was performed to assess occult synovitis and better characterize the patient’s multifactorial hand symptoms.

MRI was performed on a 3.0-T scanner using a dedicated hand/wrist surface coil. High-resolution small–field-of-view imaging was obtained with thin-section multiplanar sequences, including axial and coronal T1-weighted images, and fat-suppressed T2-weighted sequences. Images were acquired with a slice thickness of approximately 3 mm. Fat suppression was achieved using frequency-selective fat-saturation techniques. Following intravenous gadolinium administration (0.1 mmol/kg), fat-suppressed T1-weighted post-contrast images were acquired in axial and/or coronal planes to evaluate enhancement of the palmar fascia, tendon sheath, and A1 pulley.

MRI demonstrated low-signal cord-like thickening within the palmar subcutaneous tissues superficial to the flexor tendon sheaths at the level of the second through fourth metacarpal necks. These cords exhibited marked post-contrast enhancement, consistent with early Dupuytren’s disease (Fig. 1).

**Fig. 1:**
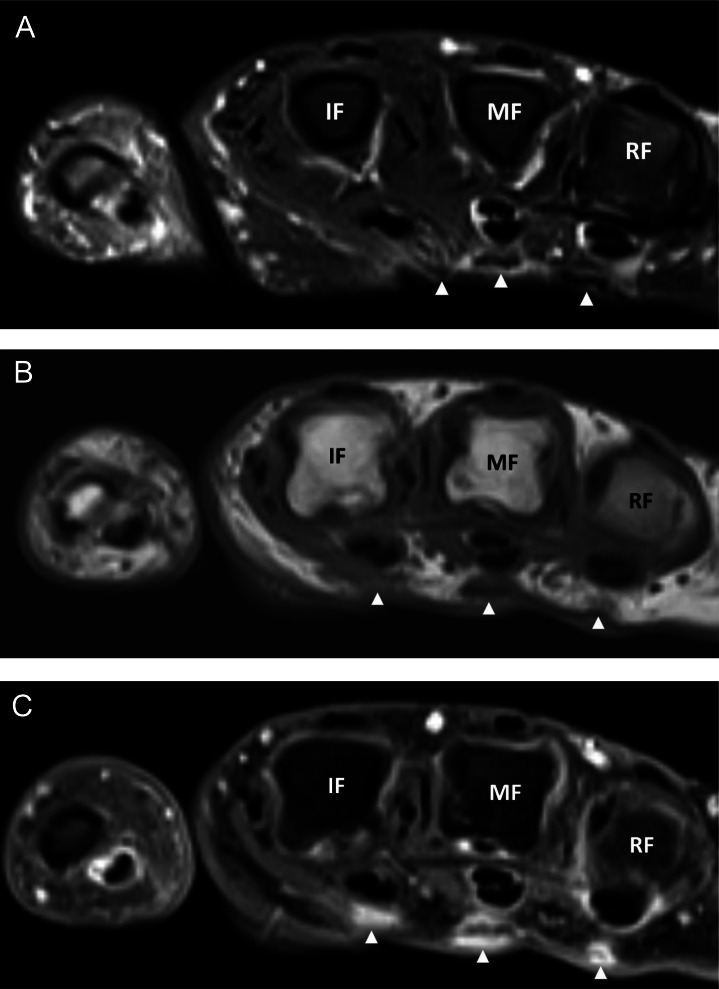
(A–C): T2-weighted axial image (A) demonstrates low-signal, cord-like structures (arrow heads) within the midline palmar subcutaneous fat of the middle finger (MF) and, to a lesser extent, the ring (RF) and index fingers (IF), located between the flexor tendons and the skin. On the pre-contrast T1-weighted image (B), these structures exhibit intermediate signal intensity and show marked enhancement on the post-contrast image (C).

Additionally, the A1 pulleys of the thumb, index, and middle fingers were thickened measuring up to 1.6 mm and demonstrated enhancement. Mild to moderate associated flexor pollicis longus tendinosis and tenosynovial enhancement were present, consistent with stenosing flexor tenosynovitis. One of the notable imaging findings in this case was the close anatomic proximity and overlapping enhancement between the palmar fascial abnormalities and the adjacent A1 pulley/tendon sheath structures on post-contrast images (Fig. 2). Coronal and axial contrast-enhanced images further illustrated the spatial relationship between the enhancing palmar fascial cords and adjacent flexor tendon sheath structures (Fig. 3).

**Fig. 2:**
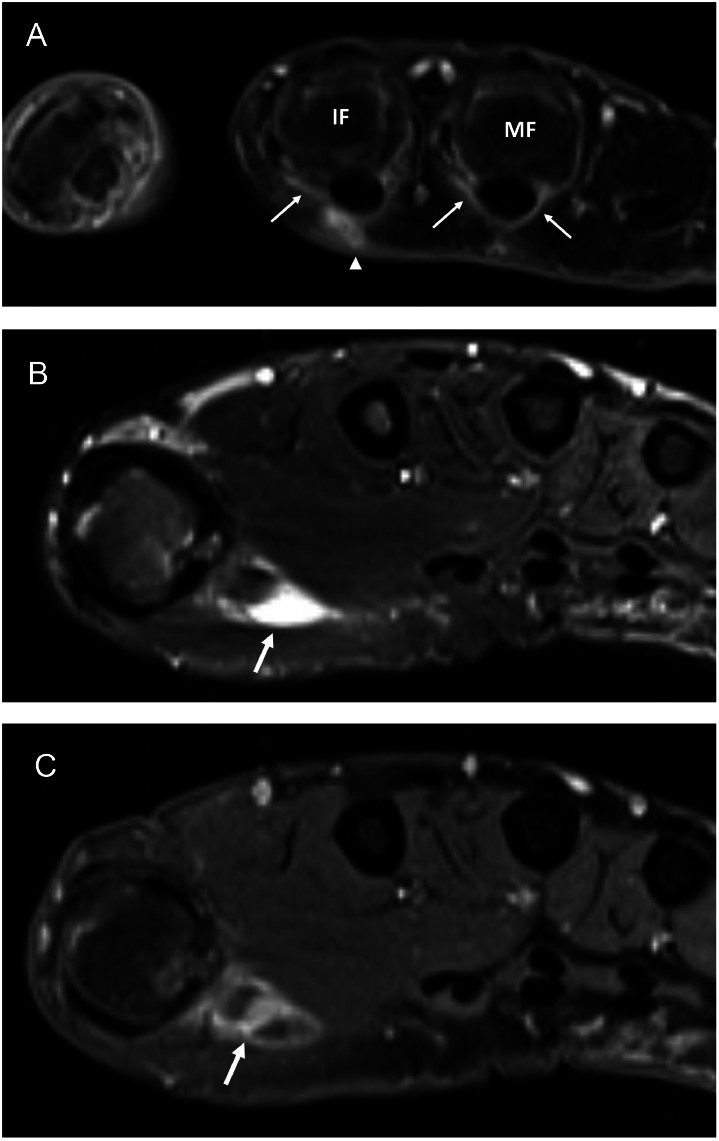
(A–C): Axial fat-suppressed T1-weighted post-contrast image (A) shows thickening and enhancement of the A1 pulleys of the index (IF) and middle fingers (MF) (arrows). Note superficial palmar extension of inflammation along the pulley (arrowhead). Axial fat-suppressed T2-weighted image (B) shows tendinosis and stenosing tenosynovitis of the flexor tendon of the thumb (arrow), which demonstrates marked enhancement on the corresponding post-contrast image (C).

**Fig. 3:**
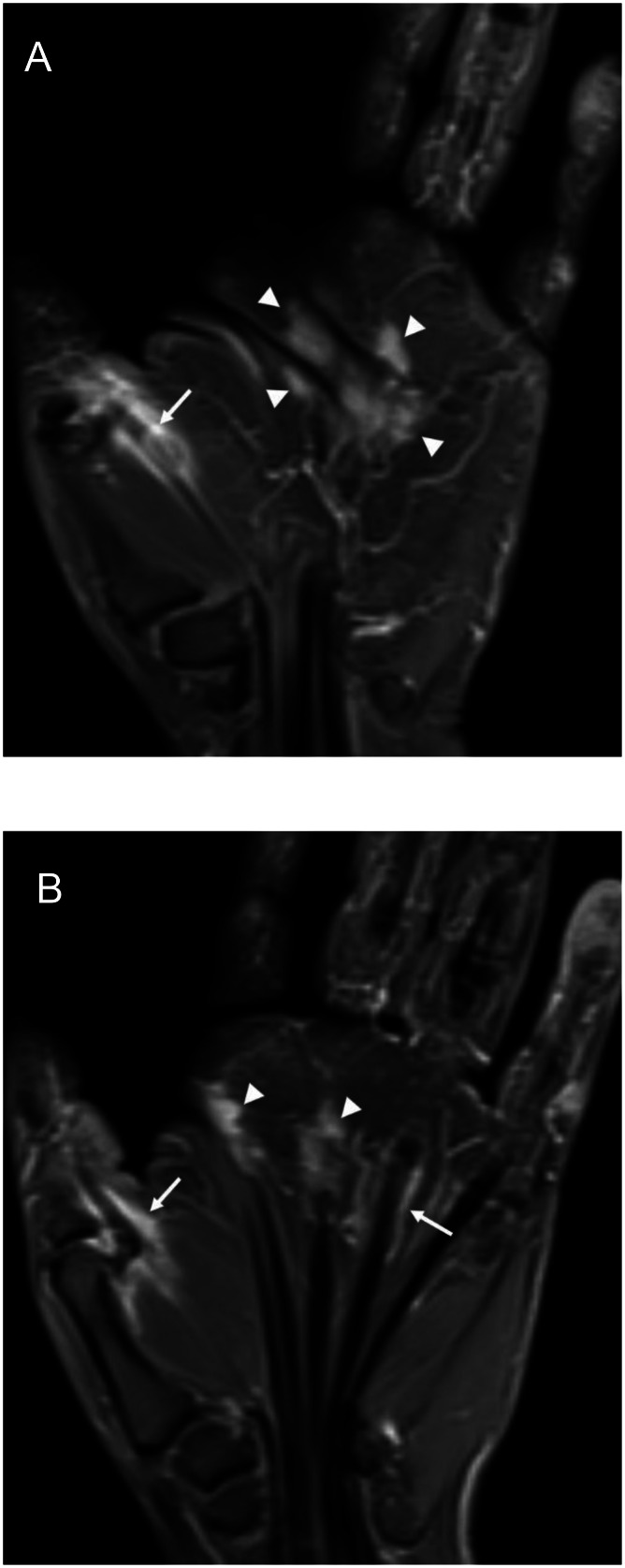
(A, B): Consecutive coronal fat-suppressed T1-weighted post-contrast images demonstrate findings at superficial (A) and deeper (B) levels of the palm. (A) The superficial image shows cord-like areas of nodular enhancement within the subcutaneous tissues of the distal palm (arrowheads). (B) The deeper image shows extension of this enhancement (arrowheads) toward the flexor tendons, with associated tendon sheath enhancement (arrows), suggestive of tenosynovitis.

The patient underwent corticosteroid injection for trigger finger and was referred for hand surgery evaluation. At 6-week clinical follow-up, triggering symptoms of the right thumb had improved with reduced locking and pain. The palmar nodules remained clinically stable and were scheduled for continued clinical monitoring without additional interval imaging.

## Discussion

Dupuytren’s disease typically manifests on MRI as focal or cord-like thickening of the superficial palmar fascia. Signal characteristics reflect tissue composition: mature fibrotic cords are usually low signal intensity on both T1- and T2-weighted sequences due to dense collagen, whereas more cellular or active lesions may demonstrate intermediate signal and enhancement [6,7] In our patient, MRI demonstrated similar findings, characterized by low-signal, cord-like thickening within the palmar subcutaneous tissues with marked enhancement, suggesting an active or early stage of the disease. Additionally, the absence of fixed contracture and a negative tabletop test were clinically consistent with an early-stage presentation.

Trigger finger is characterized by focal thickening of the A1 pulley, which normally measures less than 1 mm in thickness. Symptomatic cases often demonstrate pulley thickening (commonly >1 mm), associated enhancement, tendon signal alteration, and tenosynovial fluid [8]. Clinically, advanced stenosing tenosynovitis may produce functional limitation that can resemble fixed contracture [9]. Similarly, our case showed thickening and enhancement of the A1 pulleys of the thumb, index, and middle fingers, with associated flexor pollicis longus tendinosis and tenosynovial enhancement. These findings support a stenosing mechanism rather than isolated tendinopathy.

Both conditions, Dupuytren’s disease and trigger finger, represent fibroproliferative processes and share common risk factors, including diabetes mellitus. In this case, MRI demonstrated concurrent palmar fascial cord formation and A1 pulley pathology. The spatial proximity of these structures may contribute to overlapping symptoms and raises the possibility of a mechanical or inflammatory interaction, although the exact relationship remains speculative.

MRI offers comprehensive evaluation of the palmar fascia, pulley system, and flexor tendons in a single examination. Recognizing concurrent pathology is clinically relevant, as management differs: trigger finger is often treated with corticosteroid injection, whereas Dupuytren’s disease may be managed with observation, collagenase injection, or surgical intervention depending on severity and progression [9,10].

## Conclusion

This case demonstrates simultaneous MRI findings of Dupuytren’s disease and stenosing flexor tenosynovitis in a single patient. Awareness of potential coexistence is important when evaluating multifactorial hand symptoms. MRI enables detailed assessment of the palmar fascia and flexor tendon apparatus, facilitating accurate diagnosis and appropriate management.

## Patient consent

Written informed consent for publication was obtained from the patient/representative.

## References

[1] Rayan G.M. (2007). Dupuytren disease: anatomy, pathology, presentation, and treatment. J Bone Joint Surg Am.

[2] Vuillemin V., Guerini H., Bard H., Morvan G. (2012). Stenosing tenosynovitis. J Ultrasound.

[3] Makkouk A.H., Oetgen M.E., Swigart C.R., Dodds S.D. (2008). Trigger finger: etiology, evaluation, and treatment. Curr Rev Musculoskelet Med.

[4] Gehring M.B., Constantine R.S., Le E.L.H., Wolfe B., Greyson M.A., Iorio M.L. (2023). Analysis of a national database investigating development of trigger finger after treatment of Dupuytren disease. Plast Reconstr Surg Glob Open.

[5] Chang E.Y., Chen K.C., Chung C.B. (2015). MR imaging findings of trigger thumb. Skeletal Radiol.

[6] Yacoe M.E., Bergman A.G., Ladd A.L., Hellman B.H. (1993). Dupuytren's contracture: MR imaging findings and correlation between MR signal intensity and cellularity of lesions. AJR Am J Roentgenol.

[7] Molenkamp S., van Straalen R.J.M., Werker P.M.N., Broekstra D.C. (2019). Imaging for Dupuytren disease: a systematic review of the literature. BMC Musculoskelet Disord.

[8] Petchprapa C.N., Vaswani D. (2019). MRI of the fingers: an update. AJR Am J Roentgenol.

[9] Ryzewicz M., Wolf J.M. (2006). Trigger digits: principles, management, and complications. J Hand Surg Am.

[10] Sood A., Therattil P.J., Kim H.J., Lee E.S. (2015). Corticosteroid injection in the management of Dupuytren nodules: a review of the literature. Eplasty.

